# Cytoplasmic genome substitution in wheat affects the nuclear-cytoplasmic cross-talk leading to transcript and metabolite alterations

**DOI:** 10.1186/1471-2164-14-868

**Published:** 2013-12-10

**Authors:** Cristina Crosatti, Lydia Quansah, Caterina Maré, Lorenzo Giusti, Enrica Roncaglia, Sergio G Atienza, Luigi Cattivelli, Aaron Fait

**Affiliations:** 1 Jacob Blaustein Institutes for Desert Research, French Associates Institute for Agriculture and Biotechnology of Drylands, Ben-Gurion University of the Negev, Midreshet Ben-Gurion, 84990 Sde Boqer, Israel; 2 Consiglio per la Ricerca e la Sperimentazione in Agricoltura -Genomics Research Centre, via S. Protaso 302, 29017 Fiorenzuola d’ Arda, (PC), Italy; 3 Departamento de Mejora Genética, IAS-CSIC, Apdo. 4084, Cordoba 14980, Spain; 4 Center for Genome Research, Biomedical Sciences Department, Biological Chemistry Section, University of Modena and Reggio Emilia, Via G. Campi 287, 41125 Modena, Italy

**Keywords:** Wheat, Alloplasmic line, Nuclear-cytoplasmic interaction, ROS signaling, Cytoplasmic male sterility

## Abstract

**Background:**

Alloplasmic lines provide a unique tool to study nuclear-cytoplasmic interactions. Three alloplasmic lines, with nuclear genomes from *Triticum aestivum* and harboring cytoplasm from *Aegilops uniaristata, Aegilops tauschii* and *Hordeum chilense,* were investigated by transcript and metabolite profiling to identify the effects of cytoplasmic substitution on nuclear-cytoplasmic signaling mechanisms.

**Results:**

In combining the wheat nuclear genome with a cytoplasm of *H. chilense,* 540 genes were significantly altered, whereas 11 and 28 genes were significantly changed in the alloplasmic lines carrying the cytoplasm of *Ae. uniaristata* or *Ae. tauschii*, respectively. We identified the RNA maturation-related process as one of the most sensitive to a perturbation of the nuclear-cytoplasmic interaction. Several key components of the ROS chloroplast retrograde signaling, together with the up-regulation of the ROS scavenging system, showed that changes in the chloroplast genome have a direct impact on nuclear-cytoplasmic cross-talk. Remarkably, the *H. chilense* alloplasmic line down-regulated some genes involved in the determination of cytoplasmic male sterility without expressing the male sterility phenotype. Metabolic profiling showed a comparable response of the central metabolism of the alloplasmic and euplasmic lines to light, while exposing larger metabolite alterations in the *H. chilense* alloplasmic line as compared with the *Aegilops* lines, in agreement with the transcriptomic data. Several stress-related metabolites, remarkably raffinose, were altered in content in the *H. chilense* alloplasmic line when exposed to high light, while amino acids, as well as organic acids were significantly decreased. Alterations in the levels of transcript, related to raffinose, and the photorespiration-related metabolisms were associated with changes in the level of related metabolites.

**Conclusion:**

The replacement of a wheat cytoplasm with the cytoplasm of a related species affects the nuclear-cytoplasmic cross-talk leading to transcript and metabolite alterations. The extent of these modifications was limited in the alloplasmic lines with *Aegilops* cytoplasm, and more evident in the alloplasmic line with *H. chilense* cytoplasm. We consider that, this finding might be linked to the phylogenetic distance of the genomes.

## Background

The genetic information of the eukaryotic organisms is divided into a nuclear genome and cytoplasmic genomes (mitochondrion and chloroplast, sometimes referred to as plasmon). Nevertheless, mitochondrion and chloroplast contain only a few hundred genes, and their functionality is largely dependent on nuclear genes. This condition imposes a complex coordination between nuclear and cytoplasmic gene expression activities to assure that all mitochondrion and chloroplast proteins are timely and correctly formed [1,2]. Alloplasmic lines, alien cytoplasm substitution [3], offer a unique opportunity to study the nuclear-cytoplasmic interactions and the traits whose expression is dependent on the coordinate activity of nuclear and cytoplasmic genes [4-6]. The most relevant cytoplasm-inherited trait is cytoplasmic male sterility (CMS) [1], although other traits are also known (e.g., insect resistance [7]).

Alloplasmic lines created using a nuclear genome from *Triticum spp*. and a cytoplasm from *Aegilops* were the first ones created in grasses [8]. Nevertheless, the instability of some of the traits and a detrimental phenotype reported in the *Aegilops*-*Triticum* system has fostered the investigation of new cytoplasmic-nuclear systems. Due to its high crossability with other members of the *Triticeae* tribe [9], *Hordeum chilense* Roem et Schultz has been of interest as a source of new traits to be transferred to wheat, and it has also been suggested as a basis for new cytoplasmic-nuclear systems [10-12]. Aside from the possible beneficial effect that can be derived from the introgression of cytoplasm genomes from wild species into crops, a detrimental effect has also been consistently reported up to this point. It was observed that the alloplasmic lines carrying the *Aegilops spp*. or *H. chilense* cytoplasm performed worse than the euplasmic line in regard to many agronomic traits [6,13].

A non-natural combination of nuclear and cytoplasm genomes results in an essential alteration of the gene balance that infringes on the adaptability of the new genotype and brings about changes in quantitative traits and biological functions under the influence of mitochondrial and chloroplast genomes [14], which, in turn, can have an adverse effect on the responses to biotic and abiotic stresses [15]. The chloroplast and mitochondrial genomes work in coordination with the nuclear genome to support their functions and their adaptation to the changeable environmental conditions [16]. Conversely, alteration of the cytoplasm genome has a relevant impact on nuclear gene expression. For instance, many nuclear genes were found altered in a *Brassica napus* CMS line, particularly those encoding proteins involved in the organelles protein import machinery and the genes expressed in stamens and pollens, as well as the genes implicated in cell-wall remodeling or architecture [17]. Although substantial work has been carried out to investigate gene expression in alloplasmic lines [18-20], a majority of these studies were devoted to genes associated with floral organ development in CMS *Brassicaceae*[21]. As such, the absence of published comparative studies investigating the alteration in transcript and metabolite profiles, due to a foreign cytoplasm, limits the understanding of nuclear-cytoplasmic interaction and its output on the cellular processes. The central role of the chloroplast and mitochondria on plant cell metabolism and the known potential of CMS in crop breeding call for a comprehensive understanding of the global changes occurring as a result of foreign cytoplasm integration.

Very often the analysis of nuclear-cytoplasmic interaction is carried out by means of nuclear mutants [22], on the contrary, differences detectable between an alloplasmic line *vs*. the corresponding euplasmic can reveal the impact of small variations of the cytoplasm genome on the nuclear-cytoplasmic cross-talk. This study was carried out to assess the impact of alloplasmic lines on cell functionality. Parallel transcript and metabolite profiles were performed on three wheat alloplasmic lines obtained by cytoplasmic genome introgression from wild species (*Ae. uniaristata, Ae. tauschii* and *H. chilense*) into cultivated wheat and in their respective euplasmic genotypes. The effect that phylogenetic relatedness has on gene expression and metabolic regulation of common wheat is discussed. We hypothesize that, in order to maintain its functionality, the cell must integrate the perturbation caused by a non-natural nuclear-cytoplasmic interaction and reorganize its regulatory system and metabolism accordingly.

## Results

### The substitution of a wheat cytoplasm with an alien cytoplasms has an impact on the nuclear transcriptome

**Figure 1:**
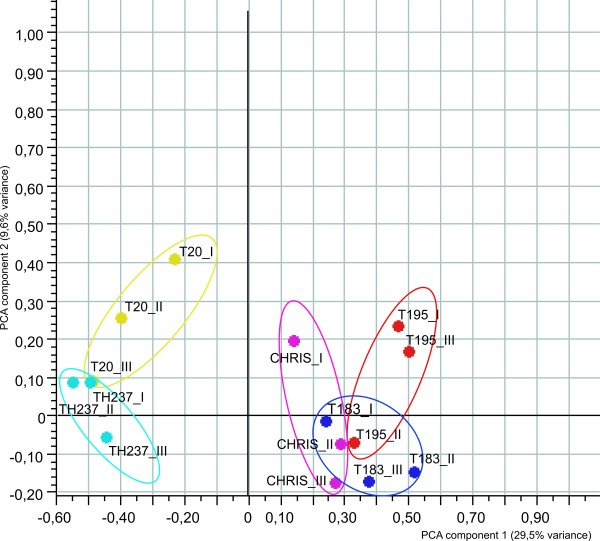
**PCA plots of wheat alloplasmic array hybridization data.** The samples have been represented according to the variance explained by the two principal component (29.5% and 9.6% on x and y axis, respectively) variations detected through transcriptome comparison. Biological replicates of each sample are surrounded by lines of different colors.TH237: alloplasmic line with *H. chilense* cytoplasm; T20: wheat accession representing the euplasmic control for TH237; T183: alloplasmic line with *Ae. uniaristata* cytoplasm; T195: alloplasmic line with *Ae. tauschii* cytoplasm; Chris: wheat cultivar representing the euplasmic control for T183 and T195.

Three wheat alloplasmic lines and the corresponding euplasmic controls were subjected to transcriptome analysis to assess the effect of cytoplasm substitution on gene expression. The alloplasmic lines T183 and T195 were developed through the introgression of the cytoplasm from *Ae. uniaristata* (T183) and *Ae. tauschii* (T195) in the nuclear background of *T. aestivum* cv. Chris by Prof. S.S. Maan [23]. The alloplasmic line TH237 was produced by introgressing the *H. chilense* accession H7 cytoplasm into the nuclear background of *T. aestivum* accession T20 [13]. The seeds of the alloplasmic lines were multiplied at IAS-CSIC and verified for cytoplasm identity with specific cytoplasm markers. T183, T195 and TH237 were selected, based on published data [13,24], in order to represent the largest cytoplasmic diversity compatible with an overall normal phenotype (no evidence of development or flower abnormalities). A global gene expression analysis was performed on RNA samples isolated from the fully expanded leaves of two-week old plants grown at 600 μE m^-2^ s^-1^. Array hybridizations were carried out using the Affymetrix Wheat Genome Array. A Principal Component Analysis (PCA, a mathematical representation of the dataset diversity [25]), performed on the transcriptome dataset, identified two main components that explain 29.5% and 9.6% of the variance (Figure 1). The first component separated the alloplasmic lines developed in the genetic background of the accession T20 from those developed in the genetic background of the cultivar Chris. The second component allowed for the separation of the alloplasmic line TH237 (*H. chilense* cytoplasm) from the corresponding euplasmic, but not the separation of the other two alloplasmic lines (*Aegilops* spp. cytoplasm) from the cv. Chris. The PCA results suggest the existence of important differences between the two genetic backgrounds T20 and Chris; therefore, all the findings of this work are based only on the comparisons between each alloplasmic line and its corresponding euplasmic.

**Figure 2:**
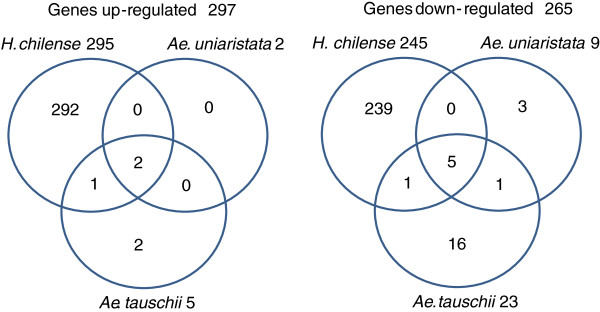
**Representation of up- and down-regulated genes.** The Venn diagrams show the genes up- and down-regulated in the alloplasmic lines developed with the cytoplasm of *Ae. uniaristata* (T183), *Ae. tauschii* (T195) and *H. chilense* (TH237) in comparison with the corresponding euplasmic controls. A single gene, up-regulated in some comparisons and down-regulated in others, has been excluded in this analysis.

A Welch t-test, using a two-fold change cut-off and a false discovery rate correction for multiple testing, identified 562 probe sets, each of them representing a putative gene, differentially modulated in at least one experimental comparison (Additional file 1: Table S1). When the wheat nuclear genome was combined with the cytoplasm of *H. chilense,* 540 genes significantly altered their expression (295 up- and 245 down-regulated), whereas 11 (2 up- and 9 down-regulated) and 28 (5 up- and 23 down-regulated) genes were significantly modulated in the alloplasmic lines carrying the cytoplasm of *Ae. uniaristata* and *Ae.tauschii*, respectively. Two up-regulated (Ta.24245.1.A1_at and TaAffx.57297.1.S1_at annotated as maturase with chloroplast and mitochondrial localization) and five down-regulated genes (TaAffx.6196.2.S1_s_at and TaAffx.6196.1.S1_atannotated as cyt b_559_, TaAffx.128795.10.S1_x_at annotated as ribosomal protein rps12, TaAffx.128617.1.S1_x_at annotated as NADPH-quinone oxidoreductase and TaAffx.128795.12.S1_x_atannotated as ABC transporter permease) were common in all comparisons (Figure 2), underlining some common responses across all alloplasmic lines. Significantly, the five down-regulated sequences were all encoded by the chloroplast genome.

**Figure 3:**
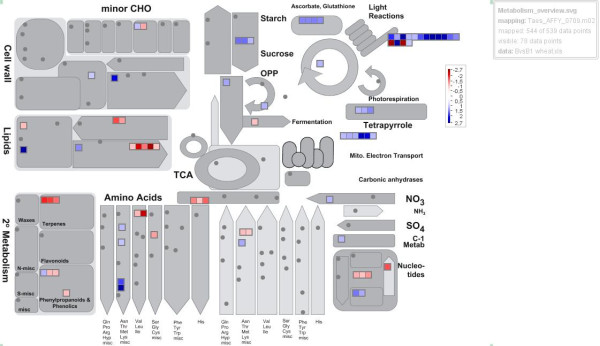
**MapMan classification of differentially expressed genes.** MapMan Metabolism overview maps showing the differences in transcript levels between the alloplasmic line TH237 (*H. chilense* cytoplasm) and the T20 euplasmic control [26]. The log_2_ expression ratios of genes altered in the comparison were loaded into the MapMan Image Annotator Module to map the wheat transcriptome data to define functional categories and to display them onto pathway diagrams. In the color scale, red and blue represent decreased and increased gene expression, respectively, in the *H. chilense* alloplasmic line as compared with the corresponding T20 euplasmic line.

The up- and down-regulated genes were divided into classes according to their expression behavior and classified using wheat gene identifier categories of the MapMan software [26]. Figure 3 summarizes the metabolism overview of up- and down-regulated genes in the *H. chilense* alloplasmic line *vs.* the corresponding euplasmic control according to the MapMan classification. The results showed that the genes associated with mitochondrial electron transport, photosynthesis, redox and hormones were generally up-regulated, while the genes associated with lipid metabolism (above all, lipid metabolism in pollen development), secondary metabolism, and signaling were mainly down-regulated.

The analysis of the sub-cellular localization of the proteins, encoded by the genes that modulated in response to cytoplasm substitution, identified 65 genes coding for plastid-targeted proteins and 22 genes coding for mitochondria-targeted proteins (15.2% of the 562 genes modulated in the experiment, Additional file 1: Table S1). Among them, 18 plastid and 11 mitochondrial-localized proteins were encoded by cytoplasmic genomes.

A validation experiment was carried out with six differentially expressed genes (*AtnMat3*, *Executer2*, *Flu*, *psbM*, *FAR*, *LSD1*, Additional file 2: Table S2) selected for their role in the chloroplast-retrograde signaling pathway (see below for their descriptions). The tested genes showed a value of 0.78 of the Pearson correlation coefficient between array and qRT-PCR expression values with an expression trend conserved across the samples between array and qRT-PCR results (detailed results are reported in Additional file 2: Table S2).

A significant proportion of the genes that were altered in response to the substitution of a wheat cytoplasm with an alien cytoplasm can be traced back to specific cellular processes or metabolic pathways. The most relevant ones are described in the next sections.

### RNA maturation-related processes in the organelles are sensitive to perturbations of the nuclear-cytoplasmic cross-talk

The nucleus controls organelle gene expression through a number of nuclear-encoded factors that act post-transcriptionally on specific organelle target transcripts (anterograde signaling pathway [27]) and that regulate the maturation, stability and/or translation of organelle transcripts. RNA maturation in mitochondrion and chloroplast, including RNA splicing, is highly dependent on nuclear-encoded splicing factors. A general consequence of the perturbation induced in the alloplasmic lines was a strong modulation of the nuclear genes coding for organelle-targeted maturases. The probe set TaAffx.57297.1.S1_at homologous to *AtnMat3*, a maturase with demonstrated mitochondrion localization [28], was up-regulated with a log_2_ fold change of 5.3, 3.7, and 1.6 in alloplasmic lines carrying *Ae. uniaristata*, *Ae. tauschii* and *H. chilense* cytoplasm, respectively. Similarly, Ta.24245.1.A1_at homologous to *AtnMat4*, localized in both mitochondrion and chloroplast [28], was also up-regulated with a log_2_ fold change of 6.5, 5.1and 3.0 in the same comparisons indicated above.

Although the targets of AtnMat3 and AtnMat4 are still unknown, it should be noted that the RNA coding for five chloroplast encoded genes, cyt b_559_ (two probe sets), ribosomal protein rps12, NADPH-quinone oxidoreductase and ABC transporter permease, were down-regulated in all alloplasmic lines, an expression trend that suggests a possibly functional link between the up-regulation of nuclear-encoded maturase genes and chloroplast expression.

Furthermore, the wheat chip array carried a probe set (TaAffx.109850.1.A1_s_at) annotated as chloroplast maturase K (MatK) in Plexdb (http://www.plexdb.org/), an essential component for the splicing of plastid RNAs. This probe set was down-regulated in the alloplasmic line carrying the *H. chilense* cytoplasm. The plastidial MatK is associated with several chloroplast genes with class II introns [29]; one of them, the mRNA precursor coding for ribosomal protein rpl2 (TaAffx.128896.8.A1_at), was also down-regulated with a log_2_ fold change of -3.3 in the same alloplasmic line.

### The substitution of a wheat cytoplasm with an alien cytoplasms alters the chloroplast retrograde signaling

To coordinate nuclear gene expression with the functional or metabolic state of plastids, the plant cells have acquired retrograde signaling pathways from plastid to nucleus, including redox signaling, Mg-protoporphyrin IX (an intermediate of the tetrapyrrole biosynthetic pathway), signals that are generated by inhibiting plastid gene expression or by accumulating various reactive oxygen species (ROS), such as hydrogen peroxide (H_2_O_2_), singlet oxygen (^1^O_2_), hydroxyl radical (OH) and superoxide anion (O_2_^-^) [30]. Excess light can potentially regulate nuclear gene expression by affecting tetrapyrrole biosynthesis. Plants exposed to high light, as was the condition for the transcriptomic experiment here reported, require specific adaptations that are mediated by chloroplast retrograde signaling [30]. Therefore, we reasoned that alterations in this signaling pathway should be evident in the experimental dataset, and a specific mining work was dedicated to finding genes that represent the key components of this signaling with an altered expression in response to a cytoplasm substitution.

*HEMA1* encodes one of the three isoforms of the glutamyl-tRNA reductase, which catalyzes the first key regulatory step of the tetrapyrrole biosynthesis, and three probe sets (Ta.3243.1.S1_at, Ta.3243.1.S1_x_at and TaAffx.8262.1.S1_x_at) corresponding to *HEMA1* were up-regulated in the alloplasmic line carrying the cytoplasm of *H. chilense*. It is known that the accumulation of chlorophyll precursors, such as Mg-Protoporphyrin IX, may stimulate the expression of the nuclear gene *HSP70*[31]. Importantly, in the *H. chilense* alloplasmic line, the up-regulation of *HEMA1* was associated with a parallel up-regulation of *HSP70-1* (Ta.24154.1.S1_s_at).

Low concentrations of ROS act as signaling molecules for a number of regulated processes during plant growth and development [32], as well as in response to a variety of environmental stimuli [33,34]. The existence of a specific ^1^O_2_ signaling pathway has been shown in *Arabidopsis* through a mutant analysis of *flu* (*fluorescent in blue light*) [35] and its regulators named *executer* (*ex1*and *ex2*) [36,37]. Three probe sets (Ta.24301.1.A1_x_at, TaAffx.128618.1.S1_at andTa.24301.1.A1_s_at), corresponding to *Flu*, and one probe set (TaAffx.110498.1.S1_at), corresponding to*Ex2,* were all up-regulated in the alloplasmic line carrying the *H. chilense* cytoplasm, in comparison with the corresponding euplasmic. Furthermore, the same alloplasmic line was characterized by an up-regulation of three probe sets (Ta.28069.1.A1_at, Ta.6594.1.S1_at and Ta.6594.1.S1_s_at), corresponding to *LSD1* (*LESION SIMULATING DISEASE 1*), a putative regulator of the hypersensitive response and light acclimation driven by ROS accumulation [38]. Notably, the up-regulation of *LSD1* was associated with an altered expression of 26 probe sets involved in the hypersensitive response to biotic stress belonging to the pathway of methyl jasmonate and salicylic acid (Table 1). Among these sequences, there were enzymes involved in the synthesis of salicylic acid (isochorismate synthases, one probe set), the WRKY70 transcription factor (two probe sets), lipoxigenase 2 (three probe sets), DIR1 (Defective Induced Resistance 1, a gene involved in the systemic acquired resistance -SAR, two probe sets), FMO1 (Flavin-dependent Monoxigenase 1, an essential component of SAR, three probe sets) and several other pathogen responsive proteins. Furthermore, in the *H. chilense* alloplasmic line, we found several down-regulated genes involved in programmed cell death: ACD1 (Accelerated Cell Dead 1, TaAffx.67544.1.S1_at), STP13 (Sugar Transport Protein 13, Ta.5766.1.S1_at), VEP1 (Vein Patterning 1, Ta.13682.1.A1_at) and two probe sets annotated as putative metacaspases (Ta.3154.1.S1_at and Ta.10581.1.A1_at) [39-42].

**Table 1:** **Probe sets encoding proteins involved in biotic stress responses significantly up- or down-regulated in the alloplasmic line TH237 carrying the *H. chilense *** **cytoplasm compared to the corresponding euplasmic line T20**

**Probe set ID**	**Log2 fold change**	**Probe set description**
Ta.24195.3.S1_at	+1.19	Vrga1-disease resistance gene homologues
Ta.24195.1.A1_at	+1.99	Vrga1-disease resistance gene homologues
TaAffx.55225.1.S1_at	+1.50	Leucine-rich repeat family protein similar to disease resistance family protein
TaAffx.94000.1.S1_at	-1.40	Barley stem rust resistance protein
Ta.21533.1.A1_at	+2.18	Powdery mildew resistance protein
Ta.21314.1.S1_at	-1.62	Disease resistance response protein (PR protein)
Ta.21314.1.S1_x_at	-1.74	Disease resistance response protein (PR protein)
Ta.22687.1.A1_at	-1.75	Disease resistance response protein (PR protein)
TaAffx.28302.2.S1_at	-1.00	Disease resistance-responsive family protein (PR protein)
TaAffx.78606.1.S1_at	-1.81	Disease resistance protein (NBS-LRR class -PR protein)
TaAffx.107480.1.S1_at	-1.61	Cold induced ice recrystallisation inhibition protein. Phytohormones involved in pathogen defense pathways (jasmonic acid and ethylene)
Ta.19859.1.S1_at	+1.36	FMO1 (FLAVIN-DEPENDENT MONOOXYGENASE 1); mono-oxygenase FMO1 is required for full expression of TIR-NB-LRR conditioned resistance to avirulent pathogens and for basal resistance to invasive virulent pathogens. Functions in an EDS1-regulated but SA-independent
TaAffx.104885.1.S1_at	+1.61	FMO1 (FLAVIN-DEPENDENT MONOOXYGENASE 1); mono-oxygenase FMO1 is required for full expression of TIR-NB-LRR conditioned resistance to avirulent pathogens and for basal resistance to invasive virulent pathogens. Functions in an EDS1-regulated but SA-independent
TaAffx.104885.2.S1_at	+1.69	FMO1 (FLAVIN-DEPENDENT MONOOXYGENASE 1); mono-oxygenase FMO1 is required for full expression of TIR-NB-LRR conditioned resistance to avirulent pathogens and for basal resistance to invasive virulent pathogens. Functions in an EDS1-regulated but SA-independent
TaAffx.131611.1.S1_at	+4.29	DIR1 (DEFECTIVE IN INDUCED RESISTANCE 1); lipid binding encodes a putative apoplastic lipid transfer protein that is involved in systemic acquired resistance (SAR). Mutants in this gene exhibit wild-type local resistance to avirulent and virulent Pseudomonas
TaAffx.131611.4.S1_at	+2.76	DIR1 (DEFECTIVE IN INDUCED RESISTANCE 1); lipid binding encodes a putative apoplastic lipid transfer protein that is involved in systemic acquired resistance (SAR). Mutants in this gene exhibit wild-type local resistance to avirulent and virulent Pseudomonas
Ta.1967.1.S1_x_at	+1.59	LOX2 (LIPOXYGENASE 2) Chloroplast lipoxygenase required for wound-induced jasmonic acid accumulation in Arabidopsis. Mutants are resistant to Staphylococcus aureus and accumulate salicylic acid upon infection. Identical to Lipoxygenase, chloroplast precursor
TaAffx.104812.1.S1_s_at	+1.63	LOX2 (LIPOXYGENASE 2) Chloroplast lipoxygenase required for wound-induced jasmonic acid accumulation in Arabidopsis. Mutants are resistant to Staphylococcus aureus and accumulate salicylic acid upon infection. Identical to Lipoxygenase, chloroplast precursor
Ta.1967.2.A1_x_at	+1.64	LOX2 (LIPOXYGENASE 2) Chloroplast lipoxygenase required for wound-induced jasmonic acid accumulation in Arabidopsis. Mutants are resistant to *Staphylococcus aureus* and accumulate salicylic acid upon infection. Identical to lipoxygenase, chloroplast precursor
Ta.8097.1.A1_at	+1.02	LOX3 (Lipoxygenase 3); iron ion binding/lipoxygenase
Ta.13650.1.A1_at	+1.93	Lipoxygenase, putative similar to LOX3 (Lipoxygenase 3), iron ion binding/lipoxygenase
Ta.21353.2.S1_at	-1.01	Putativesalicylic acid-binding protein PP
Ta.8614.1.S1_at	-1.30	WRKY70 (WRKY DNA-binding protein 70); transcription factor member of WRKY Transcription Factor; Group III. Function as activator of SA-dependent defense genes and a repressor of JA-regulated genes. WRKY70-controlled suppression of JA-signaling is partly executed by NPR1.
Ta.8614.2.S1_x_at	-1.66	WRKY70 (WRKY DNA-binding protein 70); transcription factor member of WRKY Transcription Factor; Group III. Function as activator of SA-dependent defense genes and a repressor of JA-regulated genes. WRKY70-controlled suppression of JA-signaling is partly executed by NPR1.
TaAffx.37536.1.S1_at	+1.03	Isochorismate synthases involved in salicylic acid synthesis
Ta.22221.1.S1_a_at	-1.51	ADC2 (ARGININE DECARBOXYLASE 2) encodes an arginine decarboxylase (ADC), a rate-limiting enzyme that catalyzes the first step of polyamine (PA) biosynthesis via ADC pathway in Arabidopsis thaliana.

Since *Flu, Ex* and *LSD1* are key components of the ROS chloroplast retrograde signaling [37,43,44], the identification of these genes, among those altered in the *H. chilense* alloplasmic line, as well as of other downstream genes, suggests that a change in the chloroplast genome has a direct impact on the chloroplast retrograde signaling mediated by ^1^O_2_.

### The substitution of a wheat cytoplasm with an alien cytoplasm alters the expression of photosynthetic components

Retrograde signaling is known to control the expression of many genes coding for components of the PSII, and indeed, a number of these genes, mostly encoded by the chloroplast genome, were changed in the alloplasmic lines (Table 2 and Additional file 1: Table S1). Two genes (probe sets TaAffx.6196.1.S1_at and TaAffx.6196.2.S1_s_at) coding for the cytochrome b_559_ alpha chain (psbE) were down-regulated in all alloplasmic lines, while a gene coding for a component of the psbM protein (TaAffx.48564.2.S1_s_at) was strongly down–regulated in the *H. chilense* alloplasmic line (log_2_ fold change of -7.9). Furthermore, eleven genes coding for chlorophyll a/b-binding protein (LHCII), five genes related to LT29/APX4 (a protein associated with photosystem II [45] and a gene coding for a D1 precursor (TaAffx.128757.1.S1_at) were up-regulated in the alloplasmic line carrying the *H. chilense* cytoplasm. Lastly, the alloplasmic line carrying the *Ae. tauschii* cytoplasm showed a down-regulation of a gene encoding a psbX-related protein (Ta.20878_at).

**Table 2:** Probe sets encoding components of the photosynthetic apparatus significantly up- or down-regulated in the three alloplasmic lines compared with the corresponding euplasmic line

**Probe set ID**	**Log2 fold change alloplasmic T183 vs. euplasmic Chris**	**Log2 fold change alloplasmic T195 vs. euplasmic Chris**	**Log2 fold change alloplasmic TH237 vs. euplasmic T20**	**Probe set description**
TaAffx.6196.1.S1_at	-5.81	-5.65	-5.26	Cytochromeb559 alpha chain; psbE
TaAffx.6196.2.S1_s_at	-5.92	-5.63	-4.96	Cytochromeb559 alpha chain; psbE
TaAffx.48564.2.S1_s_at			-7.93	Photosystem II M protein; psbM
Ta.20878.1.S1_at				Protein Photosystem II reaction centre X protein (PsbX), putative
Ta.22984.1.S1_x_at		-1.07		Chlorophyll a/b-binding protein (LHCII
Ta.28496.1.A1_at			+2.61	Chlorophyll a/b-binding protein (LHCII
Ta.28496.1.A1_x_a			+2.54	Chlorophyll a/b-binding protein (LHCII
Ta.29587.2.S1_x_at			+1.33	Chlorophyll a/b-binding protein (LHCII
Ta.29587.3.A1_at			+3.23	Chlorophyll a/b-binding protein (LHCII
Ta.30702.1.S1_x_at			+1.99	Chlorophyll a/b-binding protein (LHCII
Ta.3249.1.S1_at			+3.41	Chlorophyll a/b-binding protein (LHCII
Ta.3249.2.S1_x_at			+1.72	Chlorophyll a/b-binding protein (LHCII
Ta.3249.3.A1_at			+3.47	Chlorophyll a/b-binding protein (LHCII
Ta.3795.1.S1_x_at			+1.13	Chlorophyll a/b-binding protein (LHCII
TaAffx.114127.1.S1_x_at			+1.83	Chlorophyll a/b-binding protein (LHCII
Ta.488.1.S1_at			+1.50	APX4 (ASCORBATE PEROXIDASE 4)
Ta.488.1.S1_x_at			+1.51	APX4 (ASCORBATE PEROXIDASE 4)
Ta.488.2.S1_at			+1.67	APX4 (ASCORBATE PEROXIDASE 4)
Ta.488.3.S1_a_at			+1.54	APX4 (ASCORBATE PEROXIDASE 4)
Ta.488.3.S1_at			+1.48	APX4 (ASCORBATE PEROXIDASE 4)
TaAffx.128757.1.S1_at			+1.48	Photosystem II protein D1 precursor
TaAffx.128617.1.S1_x_at	-5,14	-4,65	-4.62	Chloroplast (NAD(P)H dehydrogenase, chain 5
TaAffx.128565.9.S1_at			+4.98	Protein ATPase I subunit
Ta.25398.1.S1_at			-1.10	ELIP1 (early light-inducible protein); chlorophyll binding
TaAffx.128418.14.A1_at			-1.26	ELIP1 (early light-inducible protein); chlorophyll binding
Ta.26997.1.S1_at			-1.12	Putative yearly light-inducible protein

NADH-plastoquinone oxidoreductase chain 5 (TaAffx.128617.1.S1_x_at), a component of the chloroplast NADH-ubiquinone oxidoreductase, was down-regulated in all alloplasmic lines. Furthermore, a probe set, corresponding to ATP synthase (TaAffx.128565.9.S1), was up-regulated in the alloplasmic line with the *H. chilense* cytoplasm.

To conclude, the findings described above highlight that the substitution of the wheat cytoplasm with the cytoplasm of *H. chilense*, impacts the expression of several nuclear genes encoding photosynthetic components, suggesting a modification of the nuclear-chloroplast cross-talk, at least under high light conditions.

### The substitution of a wheat cytoplasm with an alien cytoplasm modifies the expression of genes coding for components of the mitochondrial electron transport chains

The mitochondrial electron transport chain is comprised of four large protein complexes (I, II, III and IV) that interact with each other via the small lipid ubiquinone and the small protein cytochrome c [46]. Three probe sets (Ta.24230.1.S1_at, Ta.28812.1.S1_at and TaAffx.87200.1.S1_at) related to NADH dehydrogenase (EC 1.6.5.3, complex I), the first step of the oxidative phosphorylation, had an altered expression profile in the alloplasmic lines. The first two genes were up-regulated in the alloplasmic line with the *H. chilense* cytoplasm, while the last one was up-regulated in the alloplasmic lines with the *Ae. uniaristata* and the *H. chilense* cytoplasms. Furthermore, four probe sets (TaAffx.69955.1.S1_at, Ta.28701.1.A1_at, TaAffx.114431.1.S1_s_at and Ta.28645.1.S1_at), related to different subunits of the cytochrome c oxidase (COX; EC 1.9.3.1, complex IV) [45], were up-regulated in the alloplasmic line with the *H. chilense* cytoplasm. TaAffx.4544.2.S1_s_at, involved in cytochrome c oxidase biogenesis [47], was, instead, down-regulated in the *Ae. tauschii* and *H. chilense* alloplasmic lines. Notably, most of the genes described in this paragraph are encoded by the mitochondrial genome (Additional file 1: Table S1).

### The expression of genes involved in cytoplasm male sterility is dependent on the interaction between nuclear and cytoplasm genomes

The transcriptome comparison of the alloplasmic line, carrying the cytoplasm of *H. chilense* with its euplasmic control, highlighted the alteration of several genes known to be involved in the determination of cytoplasm male sterility (CMS). Three up-regulated probe sets (Ta.1967.1.S1_x_at, TaAffx.104812.1.S1_s_at and Ta.1967.2.A1_x_at) were related to LOX2 (chloroplast 13-lipoxygenase), while two down-regulated probe sets (Ta.8097.1.A1_at and Ta.13650.1.A1_at) were annotated as LOX3 (chloroplast lipoxygenase). LOX2 and LOX3 play a role in the biosynthesis of jasmonate, and both are involved in the determination of male sterility [48]. LOXes catalyze the addition of oxygen to polyunsaturated fatty acids to produce the unsaturated fatty acid hydroperoxide. Linoleic and linolenic acids are the most common substrates for LOX [49], which favors free fatty acids to sterified ones [50]. LOXes are also involved in oxylipin biosynthesis during stress, a process mainly associated with wounding and pathogen attack [51].

Lipid metabolism is essential for normal pollen development [52,53]. A starting point in fatty-acid synthesis is the conversion of acetyl-CoA into malonyl-CoA. Six down-regulated probe sets (Ta.12415.3.S1_at, Ta.23988.1.A1_at, Ta.26144.1.A1_at, Ta.26144.1.A1_s_at; TaAffx.114108.1.S1_at and TaAffx.38271.1.A1_at) are all related to fatty acyl-CoA reductase (FAR), a putative male sterility protein involved in wax biosynthesis [54]. Furthermore, a probe set (Ta.16047.1.S1_at), corresponding to *male sterility 2* (*ms2*, chloroplastic fatty acid reductase), and two probe sets (Ta.1456.1.A1_at and TaAffx.38271.1.A1_at), related to the *CER4*, both involved in the synthesis of the lipids of the outer pollen wall [55], were down-regulated in the alloplasmic line with the *H. chilense* cytoplasm; these two genes belong to a family of genes encoding FAR enzymes. In the same sample, three additional probe sets (Ta.635.2.A1_at, Ta.635.2.A1_x_at and Ta.635.3.S1_a_at), related to *POP2* (*pollen-pistil incompatibility*) [56], an enzyme of the GABA-shunt involved in the conversion of the non-protein amino acid GABA to succinyl-semi-aldehyde [57], were also down-regulated, while a probe set (Ta.20591.2.S1_a_at), related to plantacyanin involved in anther development and pollination, was up-regulated.

### GC-MS analysis reveals the impact nuclear-cytoplasmic interaction has on leaf metabolism

Considering that the nucleus-chloroplast cross-talk is essential for the adaptation of the leaf metabolism to environmental variations, GC-MS-based metabolite profiling was done on the alloplasmic lines and corresponding euplasmic controls grown at two light intensities (low light 150 μE m^-2^ s^-1^, and high light 600 μE m^-2^ s^-1^) to test if a substitution of the cytoplasm impairs light response.

**Figure 4:**
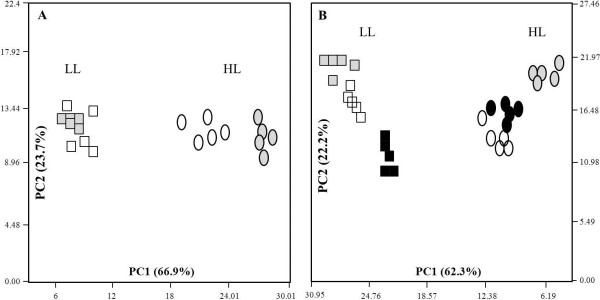
**PCA plots of the wheat alloplasmic metabolic dataset.** The metabolic dataset of leaves from plants grown at 150 (LL) and 600 (HL) μE m^-2^ s^-1^ is shown. Each spot on the plot is a representative of a sample (that is, five biological samples for each category) **A**: PCA of alloplasmic TH237 (*H. chilense* cytoplasm) *vs*. T20 euplasmic control. The rectangles and circles represent low light and high light, respectively. The white and grey colors represent T20 and TH237, respectively. **B**: PCA of alloplasmic lines T183 (*Ae. uniaristata* cytoplasm) and T195 (*Ae. tauschii* cytoplasm) in comparison with the wheat cv. Chris used as the euplasmic control. Rectangles and circles represent low light and high light, respectively. White, grey and black denote Chris, T183 and T195, respectively.

To acquire an overview of the global changes in leaf metabolism across genotypes, and conditions, the GC-MS dataset was subjected to PCA [25]. The analysis revealed a clear separation of the samples due to light effect on PC1 (Figure 4A and B) in both euplasmic backgrounds, accounting for more than 60% of the variance. These results suggest a comparable response to light intensity of the alloplasmic lines and their respective euplasmic controls, implying relatively functional mechanisms of electron dissipation and chloroplast functioning.

Nevertheless, substituting the wheat cytoplasm with the *H. chilense* cytoplasm led to a perturbation of the central metabolism in response to high light (HL), resulting in a separation of the euplasmic control from the *H. chilense* alloplasmic line with the PCA not observed under low light (LL) (Figure 4A). On the genetic background of Chris, the alloplasmic line carrying the *Ae. uniaristata* cytoplasm showed a slight separation from the euplasmic control under LL, while separating from the *Ae. tauschii* alloplasmic line. Under HL, the alloplasmic line carrying the *Ae. tauschii* cytoplasm grouped together with the euplasmic control and separated from the other alloplasmic line (Figure 4B), implying the existence of processes that affect the central metabolism in a species-specific manner.

The alteration in the metabolite profiles in response to light intensity was validated by a two-way ANOVA, separately testing the factors of cytoplasmic substitution, light treatment and their interaction (Additional file 3: Table S3 and Additional file 4: Table S4). As expected, light was confirmed as having a major impact on the metabolite profiles of all genotypes (Additional file 3: Table S3 and Additional file 4: Table S4). Substitution in T20 with the *H. chilense* cytoplasm led to the alteration in the abundance of 33% (17) of the annotated metabolites, while the interaction arising between genotype and light accounted for the change in 31% (16) of metabolites (Additional file 3: Table S3). In the Chris series, cytoplasm substitution caused a significant change in the abundance of 22% (11) and 24% (12) metabolites in alloplasmic lines with *Ae. uniaristata* and *Ae. tauschii* cytoplasm, respectively. The interaction between genotype and light treatment caused 16% (8) and 33% (17) of metabolites to be significantly altered in alloplasmic lines with *Ae. uniaristata* and *Ae. tauschii* cytoplasm, respectively (Additional file 4: Table S4). The average extent of the change in metabolite abundance was also dependent on the nuclear-cytoplasmic interaction. For example, the average fold change in the *H. chilense* cytoplasm-substituted plants was 2.6-fold, while in the Chris series, the average fold change in metabolite abundance was 0.5 and 1.9-fold in *Ae. uniaristata* and *Ae. tauschii,* respectively.

### Substitution of a wheat cytoplasm with a distant relative H. chilense cytoplasm alters leaf metabolic response to light

**Figure 5:**
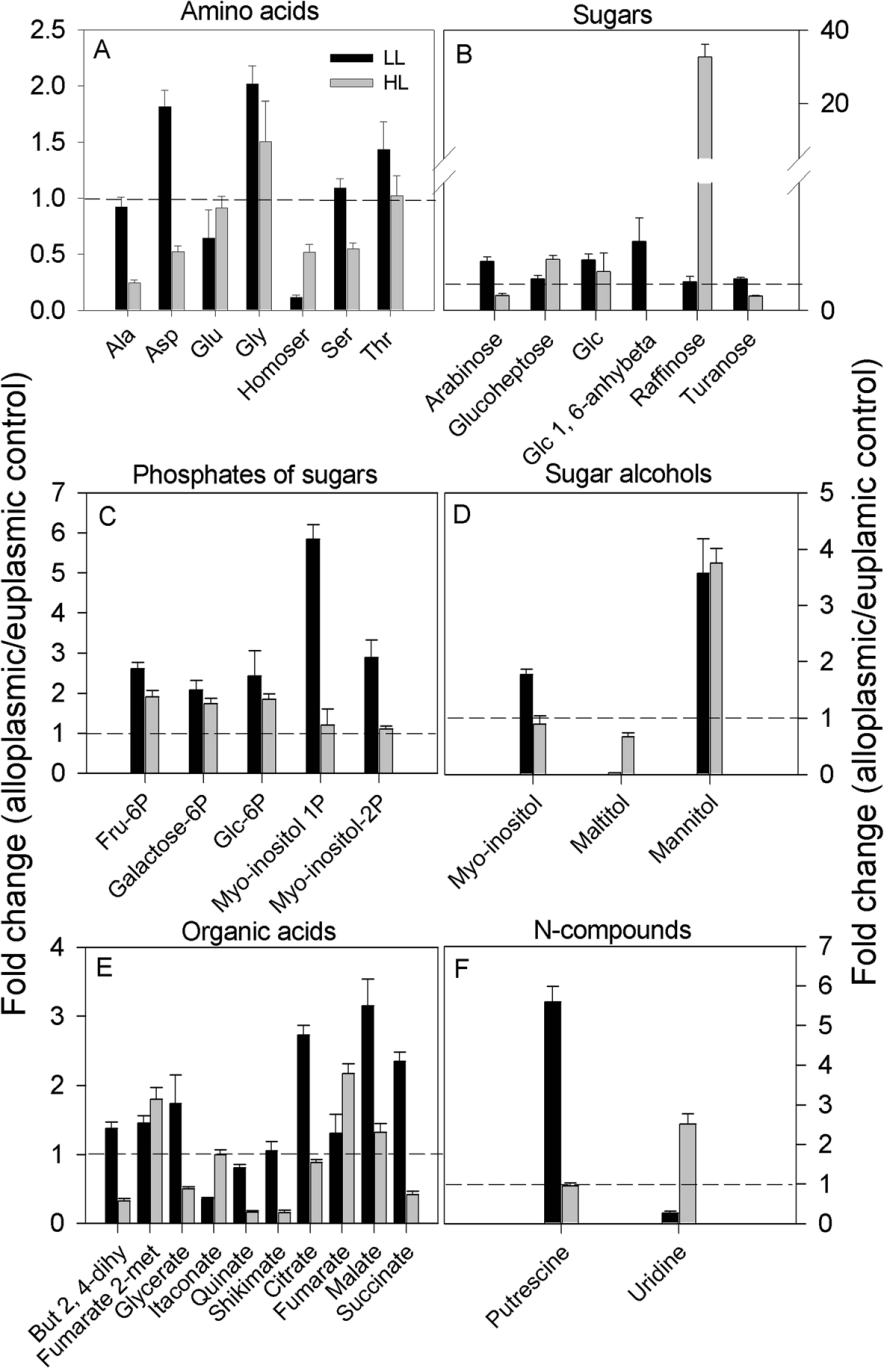
**Metabolites altered in the TH237 (*H. chilense *cytoplasm) alloplasmic line. A**-**F** denotes amino acids, sugars, sugar phosphates, sugar alcohols, organic acids, and N-compounds, respectively. Metabolites in each category that were significantly (p ≤ 0.05) altered in either low light (LL, 150 μE m^-2^ s^-1^) or high light (HL, 600 μE m^-2^ s^-1^) in the mature leaf of the alloplasmic line TH237 (*H. chilense* cytoplasm) in comparison with the wheat accession T20 used as the euplasmic control. Bars are fold changes of the alloplasmic line on the euplasmic control. The euplasmic control is given the threshold 1. Bars above or below the dashed line (threshold value 1) indicate an increase or a decrease of each metabolite in the alloplasmic genotype compared with the euplasmic control. Each bar is presented as means ± SEM of five biological repetitions. Glc, glucose; glc1, 6-anhybeta, glucose-1, 6-anhydro β.

Under LL, the comparison between the alloplasmic line carrying the cytoplasm from *H. chilense* and the corresponding euplasmic revealed significant variations in the abundance of amino acids (aspartate, glycine, glutamate and homoserine), sugars (arabinose, glucose and glucose 1, 6 anhydro-β), sugar phosphates, mannitol, myo-inositol, organic acids (mainly citrate, malate, succinate and itaconate) and putrescine (a stress-related polyamine) [58,59] (Figure 5, Additional file 5: Table S5). Markedly, in the alloplasmic line, homoserine, an intermediate in the synthesis of the aspartate-derived amino acids [60], was reduced to 10% of its content in the euplasmic control. A three-step pathway converts L-aspartate into homoserine; hence, the accumulation of aspartate and the low homoserine level might suggest a compromised step in the biosynthesis. The coordinated up-regulation of myo-inositol pathway-related genes was shown under environmental stresses [61], and often, the role of these metabolites is reported in the literature to be associated with stress, as they serve as osmoprotectants [62].

**Figure 6:**
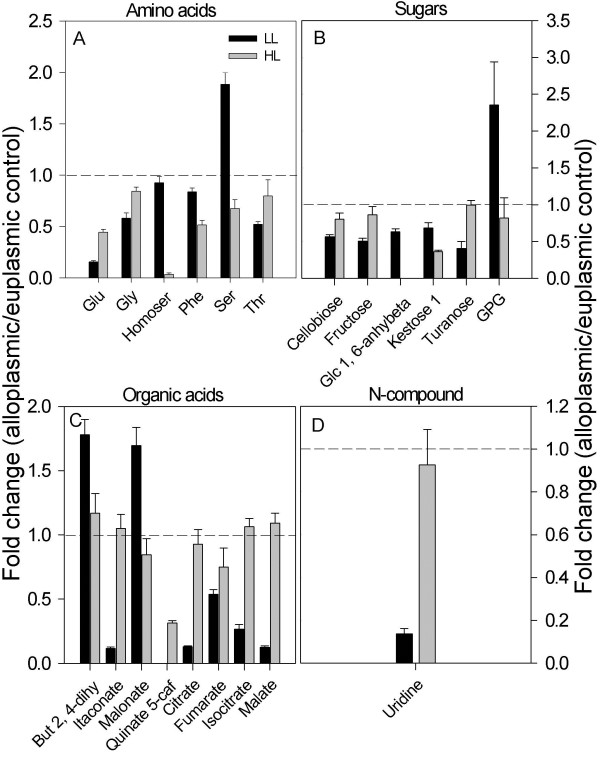
**Metabolites altered in the T183 (*Ae. uniaristata *cytoplasm) alloplasmic line. A-D** denotes amino acids, sugars, organic acids, and N-compounds, respectively Metabolites in each category that were significantly (p ≤ 0.05) altered in either low light (LL, 150 μE m^-2^ s^-1^) or high light (HL, 600 μE m^-2^ s^-1^) in the mature leaf of the alloplasmic line T183 with the *Ae. uniaristata* cytoplasm in comparison with the wheat cv. Chris used as the euplasmic control. Bars are fold changes of the alloplasmic line on the euplasmic control. The euplasmic control is given the threshold 1. Bars above or below the dashed line (threshold value 1) indicate an increase or a decrease of each metabolite of the alloplasmic genotype compared with the euplasmic control. Each bar is presented as means ± SEM of five biological repetitions. GPG, glycerophosphoglycerol.

**Figure 7:**
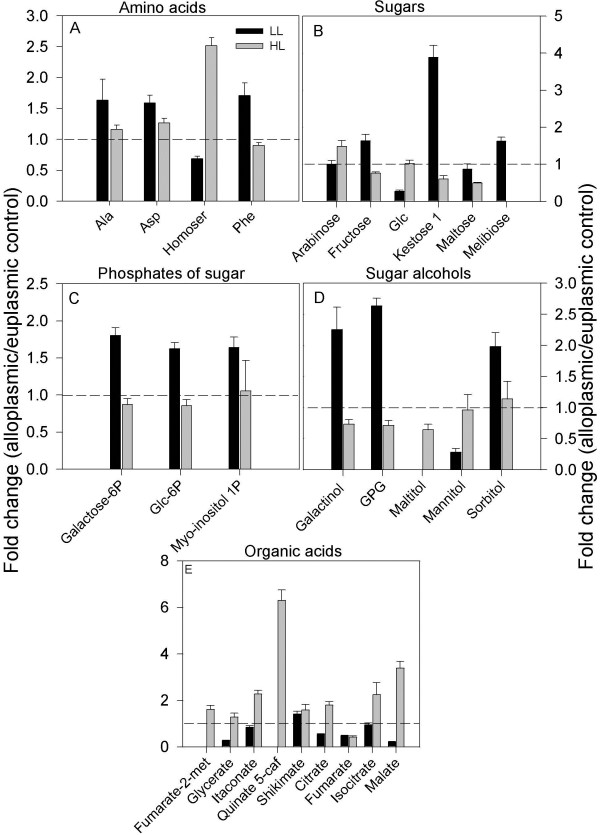
**Metabolites altered in the T195 (*Ae. tauschii *cytoplasm) alloplasmic line. A-E** denotes amino acids, sugars, sugar phosphates, sugar alcohols, and organic acids, respectively. Metabolites in each category that were significantly (p ≤ 0.05) altered in either low light (LL, 150 μE m^-2^ s^-1^) or high light (HL, 600 μE m^-2^ s^-1^) in the mature leaf of the alloplasmic line T195 (*Ae. tauschii c*ytoplasm) in comparison with the wheat cv. Chris used as the euplasmic control. Bars are fold changes of the alloplasmic line on the euplasmic control. The euplasmic control is given the threshold 1. Bars above or below the dashed line (threshold value 1) indicate an increase or a decrease of each metabolite of the alloplasmic genotype compared with the euplasmic control. Each bar is presented as means ± SEM of five biological repetitions.

*Ae. uniaristata* and *Ae. tauschii* cytoplasm substitutions showed milder effects on the metabolism of their euplasmic lines, compared to the effect of *H. chilense*. In several instances, the two *Aegilops* cytoplasms affected the metabolism in an opposite manner, while not showing significant alterations in stress-associated processes (Figures 6 and 7, Additional file 6: Table S6). Yet, a decreased level of intermediates associated with the TCA cycle (citrate, fumarate and malate) characterized both lines.

In the *Aegilops* series under HL, only a few metabolites were significantly altered, and as observed under LL, *Ae. uniaristata* and *Ae. tauschii* had opposite trends on some of the most significantly altered metabolites, e.g., homoserine and 5-caffeoylquinate (Figures 6 and 7, Additional file 6: Table S6). More significant metabolic alterations were found in the comparison between the *H. chilense* alloplasmic line and the corresponding euplasmic control under HL (Figure 5). In the *H. chilense* alloplasmic line, HL-induced alterations included a drop in the abundance of amino acid alanine, aspartic acid, homoserine and serine, compared to the euplasmic line. Significant increases in content were observed in sugars (except for arabinose and turanose), sugar phosphates and mannitol. The increase in glycine, glucose and most of the sugar phosphates in the *H. chilense* alloplasmic line could also reflect an enhancement in the photorespiratory metabolism, a finding supported by the up-regulation in the same alloplasmic line of several key genes of the photorespiration, e.g., glycolate oxidase (GOX), serine hydroxyl-methyl transferase (SHMT) and glutamine synthetase (GS). The remarkable enhancement in raffinose abundance (33-fold) and its downstream metabolite galactose is suggestive of a stress response. Under HL conditions, organic acids (butanoate-2, 4-dihydroxy, succinate, glycerate, quinate and shikimate) were at lower levels in the *H. chilense* alloplasmic line. Succinate can be produced from within the TCA cycle or by the conversion of GABA to sucinyl-semialdehyde and succinate, a reaction governed by GABA-transaminase and succinic semialdehyde-dehydrogenase; the former gene was shown to be down-regulated at the transcript level (Ta.635.2.A1_at, Ta.635.2.A1_x_at and Ta.635.3.S1_a_at). The analytical details are reported in Figure 5 and Additional file 4: Tables S4 and Additional file 5: Table S5. The decrease in shikimate intermediates, quinate and shikimate, may have supported the production of secondary metabolites.

**Figure 8:**
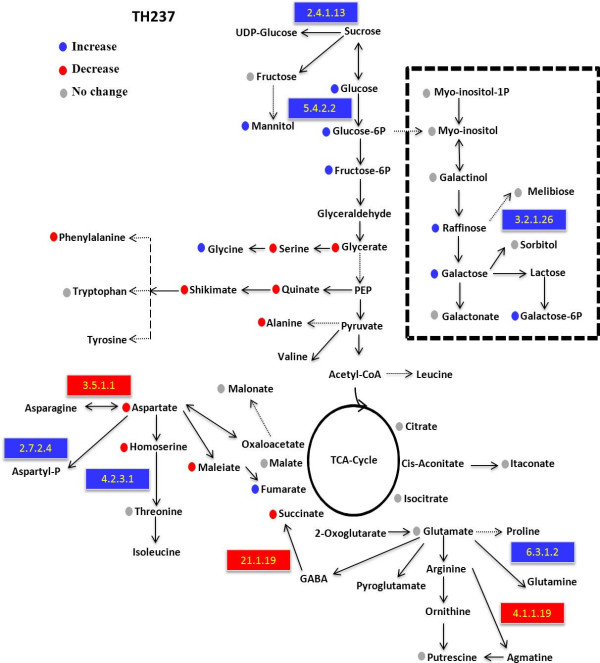
**Metabolic pathway map for the alloplasmic genotype TH237 (*H. chilense *cytoplasm) subjected to high light treatment.** Each data point is the fold change of the alloplasmic genotype on the euplasmic control. Circles and rectangles denote measured metabolites and metabolic genes, respectively. Blue, red and grey points indicate significant (p ≤ 0.05) increases, decreases and no change of metabolite and enzyme levels, respectively. Metabolic genes measured are mapped and indicated with enzyme EC numbers: arginine decarboxylase (4.1.1.19), asparaginase (3.5.1.1), glutamine synthetase (6.3.1.3), aspartyl-phosphate (2.7.2.4), threonine synthase (4.2.3.1), sucrose synthase (2.4.1.13), phospho-glucomutase (5.4.2.2), and β-fructofuranosidase (3.2.1.26).

The direct comparison between the transcriptomic and metabolomic data in the *H. chilense* alloplasmic line exposed to HL allows the identification of some correspondences between changes in the expression of genes coding for enzymes and the corresponding metabolites (Figure 8). The most clear was the up-regulation of phospho-glucomutase (EC 5.4.2.2), sucrose synthase (EC 2.4.1.13) and β-fructofuranosidase (3.2.1.26) mRNAs that were associated with an increased accumulation of sugars and sugar phosphates.

### Substitution of a wheat cytoplasm with an H. chilense cytoplasm had no effect on fatty acid accumulation

Following the alteration of enzymes involved in lipid biosynthesis in the gene expression profiling of the alloplasmic line carrying the *H. chilense* cytoplasm, a fatty acid analysis was carried out for this alloplasmic line and its euplasmic control. Eleven detectable fatty acids were annotated with linolenic acid (18:3ω3) having the highest level in the alloplasmic line and its euplasmic control, compare to the other annotated fatty acids. Nevertheless, no significant differences were detected between the alloplasmic line and the euplasmic control grown at HL (Additional file 7: Table S7). The possible explanation for the differences in the fatty acid regulation at the metabolite and gene level could be that there are yet unknown regulatory genes that may participate in a post-translational process and hence do not affect the final metabolite level.

## Discussion

The development of alloplasmic lines via alien cytoplasm substitution provides a unique tool with which to study the nuclear-cytoplasmic interactions in plants. Hexaploid wheat resulted from a combination of three nuclear genomes (A genome from *T. urartu*, B genome from *Ae. speltoides* and D genome from *Ae. tauschii*) with a cytoplasm genome from *Ae. speltoides*[63,64]. The crossability of hexaploid wheat with most *Triticeae* species has permitted the development of a panel of alloplasmic lines where the wheat nuclear genome has been coupled with cytoplasm genomes from species with different degrees of phylogenetic relatedness. The current work investigated the transcript and the metabolite profiling of three wheat alloplasmic lines carrying the cytoplasm of two very closely related species (*Ae. uniaristata* and *Ae. tauschii*) and one of a more distant species (*H. chilense*). The results showed that the *H. chilense* cytoplasm had a greater effect on the transcriptomic and metabolite profiles of its euplasmic control than did the *Aegilops* cytoplasms on theirs. It has been suggested that the negative effects produced by substituting a native cytoplasm with a cytoplasm from a more or less related species are dependent on the evolutionary distance between the two species [4,13]. These features were generally considered as the consequence of an incompatibility between nuclear and organelle-encoded components of mitochondria and/or chloroplast after their evolution into separate species. During evolutionary processes, nuclear genomes may have accumulated mutations, while organelle genomes accumulated compensatory mutations and vice versa. Therefore, the juxtaposition of cytoplasm and nucleus from distantly related genera exposes those non-compensated mutations that lead to detrimental effects on those traits determined by nuclear-cytoplasmic interactions [4]. Hence, the replacement of the wheat cytoplasm with the cytoplasm of the closely related species *Aegilops* had a limited impact on gene expression and central metabolism. In contrast, the cytoplasm of the phylogenetically more distant species *H. chilense* altered the expression of functionally relevant genes and led to impaired cellular functioning, which in turn, activated stress-related processes at the metabolite level. Nevertheless, the changes at the transcript level of the lipid biosynthetic genes were not reflected in the abundance of fatty acid. This discordance in omics data is not exclusive to this study. In fact, an extensive review by Fernie and Stitt [65] postulated that quite often, there is little linear relation between transcriptomic and metabolomic data, implying a higher level of the regulation and structure of metabolic networks. We suggest that phylogenetic relatedness infers the degree of compatibility between nucleus and cytoplasm genomes, and in turn, the co-evolution of nucleus and cytoplasm has shaped the regulatory mechanisms that control the cross-talk between the cellular genomes.

### Specific signaling mechanisms are affected by cytoplasm substitution

The present study was carried out in the fully expanded leaves of plants grown at 600 μE m^-2^ s^-1^, a condition that enables the highlighting of differences due to the activation of light-stress-associated processes known to be relevant for chloroplast and mitochondrion functionality. Since the cross-talk between nucleus and cytoplasm genomes is controlled by defined anterograde and retrograde signaling mechanisms [66], the alterations detected with transcriptomic and metabolomic analysis can be used to highlight specific signaling pathways sensitive to small variations in the cytoplasm genome.

The plastid transcripts undergo an extensive and complex maturation process that includes the splicing of introns and RNA editing which require specific proteins encoded exclusively in the nucleus. Most introns in organelles are group II introns, whose catalytic mechanism closely resembles that of the nuclear spliceosome. The plant nuclear genome contains four organelle maturase-related genes, out of the context of their evolutionary-related group-II introns dedicated to the nuclear genes [67]. All the encoded proteins are targeted to mitochondria/chloroplast [28] and act *in-trans* in the splicing of organellar-encoded introns. The finding that two (*AtnMat3* and *AtnMat4*) out of four nuclear maturase-related genes were up-regulated in all alloplasmic lines identifies the anterograde signaling that controls the RNA-maturation-related processes as one of the most sensitive to a perturbation of the nuclear-cytoplasmic interaction. Although no specific targets have been demonstrated so far for *AtnMat3* and *AtnMat4*, correlative evidence supporting a defective maturation process for some cytoplasmic-encoded genes can be found in the down-regulation of five chloroplast-encoded genes in all alloplasmic lines, a trend opposite to the maturase up-regulated expression.

Although no chloroplast retrograde pathway is well-understood mechanistically, several signals of various natures have been reported to trigger retrograde signaling from chloroplasts to nucleus, among them the signals generated by ROS accumulation. The different forms of ROS cause similar cellular damages, but each of them is involved in the activation of a specific signaling pathway [30]. High light conditions, as those employed in this work, are known to generate ROS, and consequently, the results from our study identifying a number of modifications can be linked to ROS-mediated retrograde signaling pathways. Light acclimation processes in plants act to dissipate the excess excitation energy and optimize photosynthesis under variable light intensity. Under high light conditions, the excitation energy might not be sufficiently quenched by the PSII reaction center or by carotenoids, promoting the formation of the triplet-excited state of the chlorophyll and leading to the generation of ^1^O_2_[68,69]. We found that the retrograde signaling pathway triggered by ^1^O_2_ was specifically altered in the alloplasmic line carrying the *H. chilense* cytoplasm. Transcriptomic analysis revealed an up-regulation of *flu* and *ex2*, two of the key genes identified as essential and specific components of the ^1^O_2_ signaling.

*Flu* is a negative regulator of chlorophyll biosynthesis that prevents the accumulation of intermediate that are potentially extremely destructive when illuminated, causing a massive release of ^1^O_2_. The accumulation of ^1^O_2_ leads to rapid and selective transcriptional reprogramming and finally induces programmed cell death in *flu* plants. The primary effect of ^1^O_2_ generation in the Arabidopsis *flu* mutant is the activation of a broad range of signaling pathways known to be involved in biotic and abiotic stress responses [36,37]. *Flu* and *ex* are considered specific markers of the ^1^O_2_ pathway; therefore, their up-regulation demonstrates that a change in the chloroplast genome has a direct impact on the chloroplast retrograde signaling controlled by ^1^O_2_. The up-regulation of D1 can also be interpreted as a consequence of the production of ^1^O_2_ which, in turn, promotes the irreversible oxidation of the D1 protein [68]. In cyanobacterial cells, singlet oxygen has been shown to cause direct photo-damage to PSII and the D1 protein [70,71] and to prevent PSII repair by suppressing the elongation of the D1 protein [72].

A number of *flu*-downstream genes were also found up-regulated in the *H. chilense* alloplasmic line. *LSD1* is a key gene controlling the cross-talk between ROS and SA-dependent pathways, leading to light acclimation and pathogen defense responses [44]. The up-regulation of the *LSD1* gene, a negative regulator of cell death, in the *H. chilense* alloplasmic line was associated with the altered expression of 26 probe sets involved in the hypersensitive response to biotic stress belonging to methyl jasmonate and salicylic acid pathways (Table 1) and with the down-regulation of several genes involved in programmed cell death. These results suggest that a change in the chloroplast genome has a direct impact on the chloroplast retrograde signaling that integrates light acclimation, cell death and immune defense responses, including genes engaged in ethylene, ROS, salicylic acid, glutathione, ABA, sugar and auxin signaling.

In line with the observation reported above, several significant metabolic alterations found in the *H. chilense* alloplasmic line can be associated with a typical response to high light mediated by the ROS signal [73]. It is known that soluble sugars, especially sucrose, glucose and fructose, are involved in the responses to a number of stresses, and they act as nutrient and metabolite signaling molecules that activate specific hormone-cross-talk transduction pathways, thus resulting in important modifications of gene expression and proteomic patterns. Various metabolic reactions and regulation directly link soluble sugars with the production rates of ROS, such as mitochondrial respiration and photosynthesis regulation and, conversely, with anti-oxidative processes. A metabolic analysis of the *H. chilense* alloplasmic line showed significant increases in sugars, sugar phosphates and mannitol, with a remarkable enhancement in raffinose abundance (33-fold) and of its downstream metabolite galactose. The accumulation of raffinose is in agreement with the up-regulation of two key enzymes (sucrose synthase and β-fructofuranosidase) of the raffinose metabolism (Figure 8). These oligosaccharides are associated with a number of stresses in plants [74] e.g., drought, salt, temperature [75] and light [76]. They function mainly as osmoprotectants, and raffinose is known to play a key role in ROS scavenging and the protection of the metabolism in chloroplasts from the oxidative damage caused by HL [77]. The increased abundance of a number of polyols could further support a limited response to light of the alloplasmic line [78,79]. For example, polyols are reported to play an important role in the protection of sensitive enzymes and membranes from ROS in the cytoplasm [80]. Taken together, these results suggest that, the *H. chilense* alloplasmic line is likely impaired in electron dissipation, and thus, exposure to light leads to enhanced ROS generation and the induction of ROS-scavenging processes and the accumulation of membrane protective compounds.

### Cytoplasm male sterility-associated genes are modulated in response to cytoplasm substitution

Although the alloplasmic lines here tested were all fully male fertile, the finding that some genes known to be involved in the determination of CMS have an altered expression due to the substitution of a wheat cytoplasm with the cytoplasm of *H. chilense* further demonstrates the involvement of the nuclear-cytoplasmic cross-talk in CMS, as already reported for CMS that originated after inter-species crosses [10,81,82]. It can be suggested that in the specific *H. chilense* alloplasmic line here employed (TH237), the mechanisms leading to CMS have been only partially modified without a phenotypic effect, while a more strong de-regulation of the genes here described (or of some of them) might lead to male sterility. Indeed, the male fertility of the *H. chilense* alloplasmic lines varies depending on the *H. chilense* accession used. Some *H. chilense* accessions give full fertility, while others lead to male sterility as was the case of the H1 accession [10]. The fertility restoration of CMS in the msH1 system is caused by genes located on chromosome 6H^ch^[10,11]. Notably, ten probe sets, corresponding to genes modulated in the *H. chilense* alloplasmic line and related to CMS (*LOX2*, acyl CoA reductase, *CER4* and *POP2*), matched to *Brachypodium* genes from chromosome 3 in the region syntenic with barley chromosome 6H [83]. One of them (*LOX2*) also found a match in the barley genome zipper on chromosome 6H (http://mips.helmholtz-muenchen.de/plant/barley/gz/searchjsp/index.jsp). These genes may be good candidates by which to investigate the restoration of CMS in the msH1 system.

## Conclusions

The replacement of the wheat cytoplasm with the cytoplasm of a related species affects the nuclear-cytoplasmic cross-talk leading to transcript and metabolite alterations. The alteration of the nuclear-cytoplasmic cross-talks might reflect an impaired light response capacity that exposes the alloplasmic lines to some degree of stress, as suggested by the altered expression of ROS-responsive genes and the accumulation of stress-related metabolites. The extent of these modifications was limited in the alloplasmic lines with the *Aegilops* cytoplasm, and more relevant in the alloplasmic line with the *H. chilense* cytoplasm. We consider that, this finding might be linked to the phylogenetic distance of the genomes.

## Methods

### Plant material and growth conditions

Three alloplasmic lines (T183, T195 and TH237) were used in this work. The alloplasmic lines T183 and T195 were developed through the introgression of the cytoplasm from *Aegilops uniaristata* (T183) and *Aegilops tauschii* (T195) in the nuclear background of *Triticum aestivum* cv. Chris by Prof. S.S. Maan (North Dakota State University, USA) after 10 backcrosses as described by Busch and Maan [23]. The alloplasmic line TH237 was produced by introgressing the *Hordeum chilense* accession H7 cytoplasm into the nuclear background of *T. aestivum* accession T20 [13]. The seeds of the alloplasmic lines were multiplied at IAS-CSIC and verified for cytoplasm identity with specific cytoplasm markers. T183 and T195 were checked using the cleaved amplified polymorphic sequence (CAPS) psbE & psbF-HpaII [64] that distinguishes between the *T. aestivum* and *Aegilops* cytoplasm, while TH237 was confirmed using a ccSSR marker [84] as described by Atienza et al. [13]. The alloplasmic lines have overall normal phenotypes that differ from their euplasmic control only for a few phenotypic traits (e.g., plant height) and yielding capacity [13].

Alloplasmic line TH237 was produced by transferring the *Hordeum chilense* accession H7 cytoplasm into the nuclear background of the *Triticum aestivum* accession T20 [13]. Since *H. chilense* and wheat chromosomes do not pair during meiosis, the alloplasmic line was obtained after two backcrosses as previously reported [13]. The euplasmic backgrounds were used as the control. Fifty seeds were sown in five biological replicates for each sample in pots of 11 cm diameter and grown in controlled conditions under two light intensities, 150 and 600 μE m^-2^ s^-1^, denoted low and high light intensity, respectively, in a daily regime of 12 h light at 22°C and 12 h darkness at 15°C. Plants were bulked from each pot, and three biological replicates were used for the transcriptomics, while five biological replicates were used for the metabolic profiling. Fully expanded second leaves were collected two weeks from germination in the middle of the light period and frozen in liquid nitrogen until further analysis.

### RNA isolation and array hybridization

RNA samples from the leaves of alloplasmic and the corresponding euplasmic plants grown under 600 μE m^-2^ s^-1^ were prepared using a TRIZOL reagent according to the method published at the *Arabidopsis* Functional Genomics Consortium (http://www.arabidopsis.org/portals/masc/AFGC/RevisedAFGC/site2RnaL.htm#isolation) and further cleaned using RNeasy columns according to the Qiagen RNeasy Mini Handbook. RNA quantity and integrity were confirmed on an Agilent Bioanalyzer 2100 using the RNA 6000 nano Kit. RNA samples were processed following the AffymetrixGeneChip Expression Analysis Technical Manual (Affymetrix, Inc., Santa Clara, CA). Single-stranded, then double-stranded, cDNAs were synthesized from the poly (A) mRNA isolated from 5 μg of total RNA for each sample using the Affymetrix One-Cycle Labelling Kit and Control reagents. The resulting *ds*-cDNA was column purified and then used as a template to generate biotin-tagged cRNA from an *in vitro* transcription reaction (IVT), using the Affymetrix Gene-Chip IVT Labelling Kit. The resulting biotin-tagged cRNA was fragmented and then hybridized at 45°C for 16 h (Affymetrix Gene-Chip Hybridization Oven 640) to the probe sets present on an Affymetrix Gene-Chip® Wheat Genome Array. The arrays were washed and then stained (SAPE, Streptavidin-phycoerythrin) on an Affymetrix Fluidics Station 450, followed by scanning with a Gene-Chip Scanner 3000.

Hybridization quality was verified using the standard Affymetrix controls, and all hybridizations showed the expected checkerboard pictures. The collected data were normalized using an RMA algorithm. The average background was 40.07, well within recommended levels. The percentage of “present” calls ranged between 42.9% and 46.9% of the 60 K probe sets of the array. To value the quality of biological replicates, R-squared was calculated between the replicates of the same sample, and the values ranged between 0.97 and 0.99 with an average value of 0.976.

Wheat microarray design and expression profiling data are available in Plexdb (http://www.plexdb.org) as experiment TA49: “alteration in transcript level in wheat alloplasmic lines”.

### Transcript data processing and analysis

Raw intensity values were normalized by RMA [85], using the R package Affymetrix library [86]. The same library was used to run the MAS 5.0 algorithm on raw data to produce a detection call for each probe set. These detection calls (P = “present”, M = “marginal” or A = “absent”) were used to apply an initial filtering step, since genes not expressed (“absent”) represent experimental noise and can generate false positives. All probe sets that didn’t show “present” calls in all three reps of at least one sample were removed. R-squared linear correlation coefficients were computed on the RMA expression values (log_2_-transformed) for each set of biological triplicates. RMA-filtered data were imported to the Genespring GX 7.3 (Agilent Technologies) software for all subsequent analyses. Three comparisons were done to evaluate the impact of different cytoplasms on nuclear gene expression at 600 μE m^-2^ s^-1^: *Ae. tauschii* (alloplasmic line) *vs.* Chris (euplasmic control), *Ae. uniaristata* (alloplasmic line) *vs.* Chris (euplasmic control), and *H. chilense* (alloplasmic line) *vs.* T20 (euplasmic control). Differentially expressed probe sets were identified through a Welch t-test with a Benjamini and Hochberg false discovery rate correction for multiple tests [87]. Differences in gene expression were considered to be significant when the p ≤ 0.05 and the induction or repression ratio was equal to or higher than two-fold. A Principal Component Analysis (PCA), a mathematical procedure that uses orthogonal transformation to convert a set of observations of possibly correlated variables into a set of values of linearly uncorrelated variables called principal components, is often used to analyse array and metabolomic data [25]. A PCA was carried out with Genespring GX 7.3 software and employed to assess the contribution of the genetic backgrounds and of the cytoplasms on the observed variations in gene expression. The array data were analysed using a two-fold change as the cut-off, followed by an analysis for statistically significant changes using a Welch t-test and a false discovery rate correction for multiple testing [87].

Blast searches were done using a HarvEST Affymetrix Wheat1 Chip 1.50 (http://www.harvest.ucr.edu), and only the annotations of wheat probe sets with a homology level cut-off equal to or lower than E-value = e^-10^ were considered. The functional classification was based on the wheat gene identifier categories (Affymetrix Gene-Chip Wheat probe Taes_0709) reported by the MapMan 3.1.0 software (http://gabi.rzpd.de/projects/MapMan/), a user-driven tool for displaying genomics datasets on diagrams of metabolic pathways and other biological processes [26]. The log_2_ expression ratio of genes modulated in the comparison between alloplasmic line TH237 (*H. chilense* cytoplasm) and T20 euplasmic control were loaded into the MapMan Image Annotator Module to map the wheat transcriptome data into define functional categories and to display them onto pathway diagrams. In the color scale, red represents a lower gene expression and blue represents a higher gene expression in the *H. chilense* alloplasmic line, as compared with the corresponding T20 euplasmic. The complete set of genes submitted to the MapMan analysis is given in Additional file 1: Table S1.

Probe set descriptions, functional classification and mitochondrial/chloroplast localization were then verified and implemented manually and by other bioinformatics resources, such as MIPS *Arabidopsis thaliana* database (MAtDB) Functional Catalogue (FunCat) (http://mips.helmholtz-muenchen.de/proj/funcatDB) and Plexdb (plant expression database http://www.plexdb.org).

### Validation of array data with real-time reverse transcription PCR (qRT-PCR)

An array validation was carried out with six genes (*AtnMat3*, *Executer2*, *Flu*, *psbM*, *FAR*, *LSD1*, Additional file 2: Table S2), selected because they are representative of key pathways altered in the alloplasmic lines. Total RNA (400 ng) was reverse transcribed using gene specific primers with SuperScript II (Invitrogen) according to the manufacturer’s protocol. Subsequently, the cDNAs were used for q-PCR amplifications with SYBR Green fluorescence detection. The reactions (final volume of 25 μl) were set with a QuantiFast SYBR Green PCR Master Mix (Qiagen) together with forward and reverse primers (10 mM; 2.5 μl each, see Additional file 2: Table S2) and amplified using standard cycling conditions: 95°C (10 min) and 40 cycles of amplification (95°C for 30 s and 60°C for 1 min). Expression was determined for three analytical replicates. The wheat polyubiquitin gene (Ta.24299.1.S1_at) was used as a reference.

### Metabolic profiling using GC-MS-based method

Metabolite analyses from the leaves of alloplasmic and the corresponding euplasmic plants grown at 150 and 600 μE m^-2^ s^-1^ were carried out using a GC-MS-based protocol described by Lisec et al. [88] and Roesnner et al. [89] with a few modifications. Leaf tissues (100 mg) were homogenized using a ball mill pre-cooled with liquid nitrogen and extracted in 1400 μl of methanol, 60 μl of 0.2 mg ribitol ml^–1^ in methanol as an internal quantitative standard for the polar phase. The mixture was extracted for 15 min at 70°C, mixed vigorously, and centrifuged for 10 min at 14000 rpm. The supernatant was transferred to clean 2 ml tubes. In order to separate polar and non-polar metabolites, 750 μl chloroform and 1500 μl of water was added to the supernatant. After centrifugation at 4000 rpm for 15 min, 200 μl of the upper methanol/water phase was transferred to new tubes and reduced to dryness *in vacuo*. Dried samples were re-dissolved in 40 μl of 20 mg ml^–1^methoxyaminehydrochloride in pyridine and derivatized for 120 min at 37°C followed by a 30 min treatment with 70 μl MSTFA (*N*-methyl-*N*-[trimethylsilyl] trifluoroacetamide) at 37°C and 8 μl of a retention time standard mixture (3.7% [w/v] hepatonic acid, 3.7% [w/v] nonanoic acid, 3.7% [w/v] undecanoic acid, 3.7% [w/v] tridecanoic acid, 3.7% [w/v] pentadecanic acid, 7.4% [w/v] nonadeanoic acid, 7.4% [w/v] tricosanoic acid, 22.2% [w/v] heptacosanoic acid, and 55.5% [w/v] hentriacontanoic acid dissolved in 10 mg ml^–1^tetrahydrofuran) was added before trimethylsilylation. Sample volumes of 1 μl were then injected with a split ratio of 25:1, using a hot needle technique. The gas chromatography–mass spectrometry (GC-MS) system was composed of an AS 2000 auto-sampler, a GC 8000 gas chromatograph, and a Voyager quadrole mass spectrometer (Thermo-Quest, Manchester, UK). The mass spectrometer was tuned according to the manufacturer’s recommendations, using tris-(perfluorobutyl)-amine (CF43). GC was performed on a 30-m SPB-50 column with 0.25-μm film thickness (Superlco, Bellfonte, CA). The injection temperature was set at 230°C, the interface at 250°C, and the ion source adjusted to 200°C. Helium was used as the carrier gas at a flow rate of 1 ml min^–1^. The analysis was performed under the following temperature program: 5 min of isothermal heating at 70°C, followed by a 5°C min^–1^ oven temperature ramp to 310°C, and a final 1 min of heating at 310°C. The system was then temperature equilibrated for 6 min at 70°C before the injection of the next sample. Mass spectra were recorded at 2 scan sec^–1^ with a scanning range of 50 to 600 m/z. Data was analysed using Tagfinder software [90] and statistical analysis including an ANOVA, a student t-test and a principle component analysis that were performed using dedicated software, Microsoft Excel, R software environment (http://www.R-project.org; R Development Core Team, 2004) and TMEv (http://www.tm4.org/mev/) [91].

### Data analysis using principal component analysis

Principal component analysis (PCA) visualizes the dispersion of metabolites/genes and the formation of groups of co-dispersed metabolites/genes. A PCA was performed with the software package TMEv [91] for metabolite data using the default weighted covariance estimation function. PCA is an unsupervised multivariate method that allows patterns, trends, groups and outliers in a large datasets to be easily identified. The dimensionality of complex data is reduced to what are called Principal Components (PCs) that retain the maximal amount of variation within a samples’ population. For PC and average based statistical analysis, the data was log_10_ transformed and normalized to the median of the entire sample set for each metabolite before analysis. This transformation reduces the influence of outliers and increases symmetry in a dataset [25].

### Availability of supporting data

The datasets supporting the results of this article are included within the article and its additional files.

## Abbreviations

CMS: Cytoplasmic male sterility; HL: High light; LL: Low light,^1^O_2_, singlet oxygen; PCA: Principal component analysis; ROS: Reactive oxygen species; SAR: Systemic acquired resistance.

## Competing interests

The authors declare that they have no competing interests.

## Authors’ contributions

CC and LG performed the experiment and carried out all gene expression analyses; CC and CM carried out the bioinformatics analysis and interpreted the array results. LQ carried out and interpreted the metabolic analysis. ER carried out the array hybridization. SA, LC and AF participated in the design of the study. CC, LQ, SA, LC and AF wrote the manuscript. All authors read and approved the final manuscript.

## Supplementary Material

Additional file 1: Table S1 Probe sets detecting transcripts more than two-fold up-or down-regulated in the comparisons between each alloplasmic line and the corresponding euplasmic control. Click here for file

Additional file 2: Table S2 Genes selected for array validation and the corresponding primers employed in the qRT-PCR analysis. Expression data detected with microarray and qRT-PCR analysis are reported for comparison. Click here for file

Additional file 3: Table S3 Data of two-way ANOVA metabolites that significantly changed in the alloplasmic line TH237 (*H. chilense* cytoplasm) and its euplasmic control T20 due to genotype, light treatment and the interaction effect between them. Click here for file

Additional file 4: Table S4 Data of two-way ANOVA metabolites that significantly changed in the alloplasmic lines T183 (*Ae. uniaristata* cytoplasm) and T195 (*Ae. tauschii* cytoplasm) and their euplasmic control (Chris) due to genotype, light treatment and the interaction effect between them. Click here for file

Additional file 5: Table S5 Metabolite variations in the comparison between the alloplasmic line with the *H. chilense* cytoplasm (TH237) and the corresponding wheat euplasmic control (T20). Values expressed as fold change for plants grown at low light and high light. Click here for file

Additional file 6: Table S6 Metabolite variations in the comparison between the alloplasmic lines with *Ae. uniaristata* (T183) or *Ae. tauschii* (T195) and the corresponding wheat euplasmic control (Chris). Values expressed as fold change for plants grown at low light and high light. Click here for file

Additional file 7: Table S7 Fatty acids measured in the alloplasmic line TH237 (*H. chilense* cytoplasm) and its euplasmic control T20. Data was subjected to a student t-test to test for fatty acids that were significant (p ≤ 0.05) between the alloplasmic line and its euplasmic control. Click here for file
